# A High-Throughput Standard PCR-Based Genotyping Method for Determining Transgene Zygosity in Segregating Plant Populations

**DOI:** 10.3389/fpls.2017.01252

**Published:** 2017-07-24

**Authors:** Lige Geng, Dewayne D. Deng, Martin J. Wubben, Johnie N. Jenkins, Jack C. McCarty, Ibrokhim Abdurakhmonov

**Affiliations:** ^1^Hebei Center for Agriculture Genetic Resources Preservation, Institute of Cereal and Oil Crops, Hebei Academy of Agriculture and Forestry Sciences/Crop Genetics and Breeding Laboratory of Hebei Province Shijiazhuang, China; ^2^Crop Science Research Laboratory, Genetics and Sustainable Agriculture Research Unit, United States Department of Agriculture – Agricultural Research Service, Mississippi State MS, United States; ^3^Center of Genomics and Bioinformatics, Academy of Sciences of Uzbekistan Tashkent, Uzbekistan

**Keywords:** transgene, PCR, bimodal, method, plant breeding, cotton

## Abstract

In crop research programs that implement transgene-based strategies for trait improvement it is necessary to distinguish between transgene homozygous and hemizygous individuals in segregating populations. Direct methods for determining transgene zygosity are technically challenging, expensive, and require specialized equipment. In this report, we describe a standard PCR-based protocol coupled with capillary electrophoresis that can identify transgene homozygous and hemizygous individuals in a segregating population without knowledge of transgene insertion site. PCR primers were designed to amplify conserved T-DNA segments of the 35S promoter, OCS terminator, and NPTII kanamycin resistance gene in the pHellsgate-8 RNAi construct for the *Gossypium hirsutum* phytochrome A1 gene. Using an optimized multiplexed reaction mixture and an amplification program of only 10 cycles we could discriminate between transgene homozygous and hemizygous cotton control DNA samples based on PCR product peak characteristics gathered by capillary electrophoresis. The protocol was refined by evaluating segregating transgenic progeny from nine BC_1_S_1_ populations derived from crosses between the transgenic cotton parent ‘E-1-7-6’ and other cotton cultivars. OCS PCR product peak height and peak area, normalized by amplification of the native cotton gene GhUBC1, revealed clear bimodal distributions of OCS product characteristics for each BC_1_S_1_ population indicating the presence of homozygous and hemizygous clusters which was further confirmed via K-means clustering. BC_1_S_1_ plants identified as homozygous or hemizygous were self-fertilized to produce BC_1_S_2_ progeny. For the homozygous class, 19/20 BC_1_S_2_ families confirmed the homozygous BC_1_S_1_ prediction while 21/21 BC_1_S_2_ families confirmed the hemizygous prediction of the original parent. This relatively simple protocol provides a reliable, rapid, and high-throughput way of evaluating segregating transgenic populations using methods and equipment common to crop molecular breeding labs.

## Introduction

Cotton (*Gossypium* spp.) continues to be the basic resource for thousands of consumer and industrial products manufactured in the world, and its contribution to the fiber and food industry continues to grow in importance. The simultaneous improvement of fiber quality and yield in Upland cotton (*G. hirsutum* L.) has long been a challenging objective for many public and private breeding programs. In a recent study, the RNAi-mediated silencing of the *G. hirsutum* phytochrome A1 gene (PHYA1) resulted in a substantial improvement in fiber length and other fiber characteristics (Abdurakhmonov et al., 2014). In these experiments, RNAi was accomplished via expression of a PHYA1 hairpin using the pHellsgate-8 binary vector (Abdurakhmonov et al., 2014). Given the potential for this technology to dramatically ‘fast-forward’ long standing cotton improvement goals, our research group has endeavored to determine the effects of the pHellsgate-8::PHYA1 RNAi construct in a variety of genetic backgrounds that had been developed specifically for United States cotton production. To accomplish this task, a backcross breeding program was initiated whereby the transgenic event E-1-7-6 was used as a parent in crosses with nine United States cultivars. To be successful, we must be able to not only track the pHellsgate-8::PHYA1 RNAi construct in subsequent generations but also determine the homozygosity or hemizygosity of individual progeny in the BC_1_S_1_ generation and other downstream segregating generations.

A variety of methods have been developed that can distinguish between homozygous and hemizygous alleles at a particular locus. Quantitative real-time PCR (qPCR) combines amplification and detection in a single step and offers the possibility of simultaneous amplification of the segment of interest and measurement of the amount of resulting DNA molecules through reaction cycles (Heid et al., 1996). Due to its high level of sensitivity, qPCR has been successfully used to determine transgene copy number in transgenic maize (Shou et al., 2004), rice (Yang et al., 2005), and wheat (Li et al., 2004). qPCR has also been used to identify homozygous transgenic plants at a single locus and in tracking multiple transgenes (Wang et al., 2015). While the success of qPCR to track transgene zygosity has been demonstrated, the procedure itself is technically challenging and requires relatively expensive consumables. Methods similar to qPCR such as thermal asymmetric interlaced-PCR (TAIL-PCR; Liu et al., 1995) and, more recently, digital droplet PCR (ddPCR) (Hindson et al., 2011) have been used to provide information about the integration status of a transgenic allele(s) in genomes (Larkan et al., 2016). ddPCR is considered an appropriate method for zygosity testing due to its reliable and highly consistent results (Glowacka et al., 2016; Passricha et al., 2016). However, these processes are still labor intensive and less well suited for automation, which may be classified as costly procedures and/or lower throughput strategies (Zhang et al., 2015). Next generation sequencing (NGS) is advantageous because there is no need to normalize the PCR for producing robust zygosity data, which could be a tool for high-throughput transgenic genotype identification. However, this assay requires that the exact integration site of the transgene is known including adjacent genomic sequences, and it is unclear whether NGS would be suitable for tetraploid and hexaploid crops. In such cases, the PCR equalization and the normalization of reads may once again become necessary (Fritsch et al., 2015).

PCR products generated using standard amplification protocols can be detected through gel electrophoresis or capillary array electrophoresis technology. Capillary electrophoresis allows recognizing the specific PCR yield of a reaction via measurement of the peak height and/or peak area. We hypothesized that a determination of transgene zygosity in an individual could be accomplished using standard PCR of transgene-specific target sites if product yield was analyzed during the logarithmic phase of amplification. In this report, we provide an optimized protocol for determining transgene zygosity in segregating plant populations using an abbreviated standard PCR program of 10 cycles followed by product analysis via capillary electrophoresis and statistical analyses of product peak height and peak area. This simple, rapid, reliable and high-throughput technique would be accessible to the majority of crop molecular breeding laboratories.

## Materials and Methods

### Plant Materials

The RNAi-PHYA1 transgenic event ‘E-1-7-6’ was used in this study. E-1-7-6 carries the pHellsgate-8::PHYA1 RNAi construct (Abdurakhmonov et al., 2014). In addition, the *G. hirsutum* line Coker-312 (genetic background of E-1-7-6) and two transgene-null sibling lines of E-1-7-6 (Null Seg 1-7 and Null Seg 31-10) were used in this study. These lines, along with E-1-7-6, were planted in the USDA-ARS greenhouse at Mississippi State, MS for genotyping protocol development. All greenhouse and field experiments that included line E-1-7-6 or its derivatives were performed under APHIS permit number 16-049-107r.

As part of an ongoing project to determine the effects of the pHellsgate-8::PHYA1 RNAi construct in a variety of genetic backgrounds, six *G. hirsutum* cultivars and three germplasm breeding lines were used in a backcross program (**Table 1**). Two-hundred-forty individuals of each BC_1_S_1_ population along with E-1-7-6, transgene-null sibling lines, and Coker-312 were grown in the field at Mississippi State, MS for transgene genotyping. Confirmation of BC_1_S_1_ plant transgene zygosity was accomplished by testing for transgene segregation in 488 BC_1_S_2_ progenies derived from 21 hemizygous BC_1_S_1_ plants and 456 BC_1_S_2_ progenies derived from 20 homozygous BC_1_S_1_ plants.

**Table 1:** Germplasm used for back-crossing to the E-1-7-6 transgenic line.

Genotype	PI or PVP No.	Developer
Coker312	PI 529278, PVP 7200100	Coker’s Pedigreed Seed Co.
DP90	PI 529529	Delta & Pine Land Co.
SG747	PI 656375	Sure-Grow Seed, Inc.
PSC355	PI 612974	Phytogen Seed Company, LLC
FM966	PVP 200100209	Bayer CropScience Co.
UA222	PI 664929	University of Arkansas
TAM B182-33	PI 654362	Texas A&M University
MD51ne	PI 566941	William R. Meredith Jr., USDA-ARS
MD90ne	PI 634931	William R. Meredith Jr., USDA-ARS


### Genomic DNA Extraction

A single 10 mm-diameter leaf punch was taken from a young leaf and collected into a 1.5 mL centrifuge tube. DNA was extracted directly from the fresh tissue using the DNeasy 96 Plant Kit (QIAGEN, Valencia, CA, United States) per the manufacturer’s instructions with the following modifications: (i) sodium metabisulfite was added to the lysis buffer to a final concentration of 10 mM from a freshly prepared 1 M stock solution (Horne et al., 2004) and (ii) the incubation time in lysis buffer at 65°C was increased to 45 min. All DNA extractions were performed using a Microlab Star robot (Hamilton, Reno, NV, United States). Genomic DNA quality and quantity were measured using a Synergy HT plate reader with Gen5 2.06 application software (BioTek, Winooski, VT, United States). All DNA samples were normalized to a concentration of 5 ng/μL using the Microlab Star robot to serve as template in subsequent PCR (polymerase chain reaction) experiments.

### PCR Primer Design

PCR primer pair sequences were designed that targeted three regions of the pHellsgate-8::PHYA1 RNAi T-DNA sequence. These primer pairs amplified portions of the following vector components: the CaMV 35S promoter (35S_S; forward 5′-GCTCCTACAAATGCCATCATTG-3′, reverse 5′-CTTGCTTTGAAGACGTGGTTG-3′), the octopine synthase terminator (OCS_S; forward 5′-CTGCTTTAATGAGATATGCGAGAC-3′, reverse5′-CGGTAAGGATCTGAGCTACAC-3′), and the neophospho-transferase resistance gene (NPTII-3; forward 5′-CTGCCGAGAAAGTATCCATCAT-3′, reverse 5′-GATCATCCTGATCGACAAGACC-3′). All primers were designed using the PrimerQuest online tool available at www.idtdna.com. Primer sequences specific to GhUBC1 were synthesized according to Yi et al. (2008). To perform multiplex PCR, the 5′-end of all forward primers were labeled with different fluorescent dyes compatible with the ABI PRISM 3130xl Genetic Analyzer^TM^ (Applied Biosystems, Foster City, CA, United States).

### PCR for Transgene Zygosity Determination

PCR reactions for 96-well plates were performed in a total volume of 10 μL using 12.5 ng of DNA as template. For 384-well plates, a total volume of 5 μL was used along with 6.25 ng of DNA template. The final concentrations of the remaining reaction components were as follows unless otherwise noted: 0.15 μM primer, 0.05 mM each dNTP, 1x GeneAmp PCR Gold Buffer (Applied Biosystems), 1% (w/v) PVP (10,000 mw, Sigma, St. Louis, MO, United States), 0.05 units AmpliTaq Gold DNA polymerase (Applied Biosystems), and 3.0 – 4.0 mM MgCl_2_. PCR amplification was carried out using a DNA Engine Tetrad^®^2 Thermal Cycler (Bio-Rad, Hercules, CA, United States). All reactions were performed with an initial 7 min incubation at 95°C followed by a touchdown protocol of 14 cycles of 94°C for 15 s, 65°C for 30 s (decreasing by 0.7°C with each cycle), and 72°C for 1 min. After the touchdown protocol, amplification was accomplished using the following program: (94°C for 15 s, 55°C for 30 s, and 72°C for 1 min) × 5, 10, 15, 20, 30, or 40 cycles. All reactions were subjected to a final incubation at 72°C for 30 min. PCR product visualization was accomplished using the automated ABI PRISM 3130 Genetic Analyzer^TM^ according to manufacturer’s protocol on a Genetic Analyzer 3130xl. Raw data was analyzed using GeneMapper^TM^ 4.1 software (Applied Biosystems). PCR products were characterized quantitatively by the size of the amplicon in base pairs, amplicon peak height, and amplicon peak area. These characteristics (i.e., amplicons size, peak height, and peak area) are considered ‘traits’ for each primer pair used in the current study.

### Data Analysis

Peak height and area values, i.e., traits, generated by the 35S, OCS, and NPTII primer pairs were normalized using the corresponding values given by GhUBC1 amplification. GhUBC1 is a verified single copy gene in *G. hirsutum* (Zhang et al., 2003) and its trait values for a given sample would help to minimize sample-to-sample variation. Trait data normalization was conducted by the statistical conversion:

*T* = (*X*/*Y*)^∗^*Z*
Where, *T* = normalized trait value of 35S, OCS, or NPTII PCR product;
*X* = Original trait value of 35S, OCS, or NPTII PCR product;
*Y* = Original trait value of GhUBC1 PCR product;
*Z* = Population median of the trait value of GhUBC1 PCR product. The median trait value was employed because of possible non-normal distribution nature of a population.

The population distribution, trait correlation, and entry cluster analysis for all BC_1_S_1_ populations were determined using JMP Genomics 6.0 (SAS Institute, Raleigh, NC, United States). To discriminate between transgene homozygous and hemizygous individuals within each segregating BC_1_S_1_ population, two clustering approaches were employed. First, the presence of transgene homozygous and hemizygous individuals in a population would create a bimodal distribution of individuals. The valley, or ‘pit,’ separating the modes was used to group the individuals into zygosity classes. The second method used K-Means clustering with two clusters as default for multivariable analysis based on particular trait values for the 35S, OCS, or NPTII PCR products. The Tukey–Kramer HSD (honest significant difference) was used as significance test for two separated groups identified by K-Means clustering. The K-Means approach to clustering performs an iterative alternating fitting process to form the number of specified clusters and assign points to the closest clusters, which is a special case of a general approach called the EM (expectation maximization) algorithm. Due to expected unequally sized clusters, the points give preference to being assigned to the larger cluster; therefore, CCC (Cubic Clustering Criterion) was used to evaluate the model as a good fit if it was greater than or equal to 2 (Hartigan, 1985). The ‘goodness of fit’ of an observed genotypic class distribution in a BC_1_S_1_ population to the 1:2:1 ration was tested by the likelihood ratio and Pearson Chi-square tests.

## Results and Discussion

### Optimization of PCR Parameters

The optimization of the pHellsgate8-specific primer pairs 35S_S, OCS_S, and NPTII-3 centered on manipulating the following parameters: (i) final concentration of MgCl_2_ in the PCR reaction, (ii) number of amplification cycles in the PCR program, and (iii) single versus multiplex PCR. For these experiments genomic DNA from a Coker312 × E-1-7-6 F_1_ plant was used as a hemizygous transgenic control while DNA from E-1-7-6 plants served as a homozygous transgenic control. For a negative control genomic DNA from a transgenic-null sibling line of E-1-7-6 was used as template.

**FIGURE 1:**
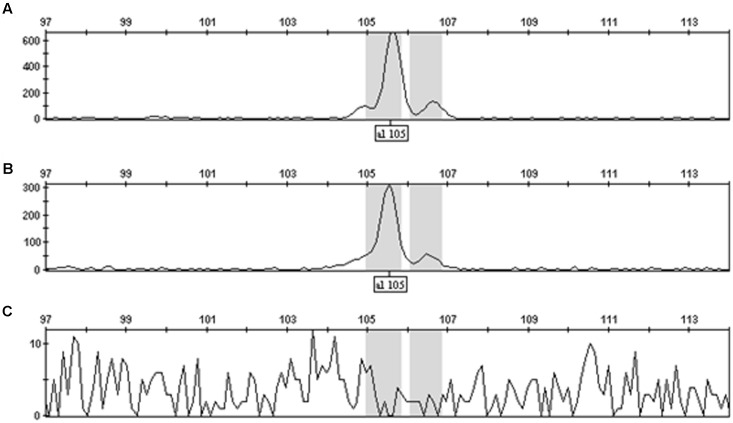
GeneMapper^TM^ display of OCS_S PCR product peak heights for plants homozygous **(A)**, hemizygous **(B)**, or null **(C)** for the pHellsgate-8::PHYA1 RNAi construct. Note the change in scale between **(A)** and **(B)**.

**FIGURE 2:**
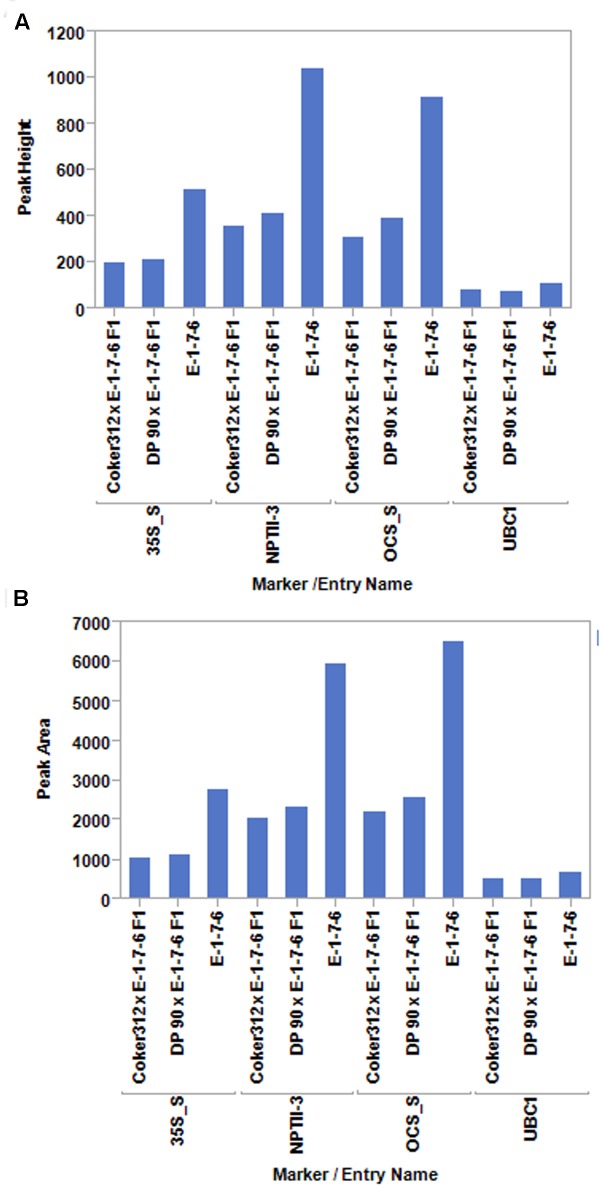
PCR product peak heights **(A)** and peak areas **(B)** for the pHellsgate-8-specific primer pairs 35S_S, NPTII-3, and OCS_S and the single copy control gene GhUBC1 (UBC1) using genomic DNA from plants homozygous (E-1-7-6) or hemizygous (F_1_) for the pHellsgate-8::PHYA1 RNAi construct in an optimized 10 cycle PCR program.

These experiments were initiated under the premise that the amplification of a target site within the pHellsgate8 T-DNA would result in differing amounts of final product being produced depending on if the T-DNA was in a hemizygous versus homozygous state. To determine the validity of this premise 35S_S, OCS_S, and NPTII-3 primers were run separately on hemizygous, homozygous, and null DNA templates using PCR programs having 5, 10, 15, 20, 30, or 40 cycles. Regardless of primer, programs having only five cycles did not produce sufficient amounts of product for detection by capillary electrophoresis (data not shown). PCR programs of 20, 30, or 40 cycles produced roughly the same amount of product regardless of primer and starting template, i.e., hemi- or homozygous. In contrast, PCR programs having 10 cycles consistently generated product amount ratios, calculated from peak height and peak area values, of ∼0.50 for hemizygous product/homozygous product for the 35S_S and OCS_S primer pairs (**Figure 1** and Supplementary Table S1). The dilution level of PCR product prior to capillary electrophoresis, ranging from undiluted to 15 × dilution, did not affect the ability of these primers to discriminate between hemi- and homozygous transgenic plants. (Supplementary Table S1). PCR parameters were further refined by testing final MgCl_2_ concentrations of 3.0, 3.5, and 4.0 mM in single reactions and in multiplex reactions where the 35S_S, OCS_S, NPTII-3, and GhUBC1 were combined. We determined that a MgCl_2_ concentration of 4.0 mM in a multiplexed reaction (all four primer pairs combined) produced optimal results (Supplementary Tables S2, S3). The finalized PCR reaction conditions (10 cycles, 4.0 mM MgCl_2_, multiplexed) were tested on another hemizygous transgenic DNA sample derived from a DP90 × E-1-7-6 F1 plant. The 35S_S, NPTII-3, and OCS_S primers each produced PCR product peak area and peak height ratios of ∼0.5 for hemizygous versus homozygous transgene genotypes (**Figure 2** and Supplementary Table S3). In contrast, the control GhUBC1 primer gave product traits that were similar regardless of starting template, thereby confirming its utility as an internal PCR control (**Figure 2** and Supplementary Table S3).

### Determination of Transgene Zygosity in Segregating BC_1_S_1_ Populations

Nine BC_1_S_1_ populations were created from crosses made between E-1-7-6 and the nine genotypes listed in **Table 1**. A total of 240 plants from each population were genotyped using the 35S_S, OCS_S, and NPTII-3 primers using the optimized PCR and electrophoresis protocol described above. The single copy internal control GhUBC1 primer was also run on each sample. Only those samples that were successfully amplified with each transgene primer were considered ‘transgenic’ and included in downstream zygosity analyses. Samples that produced a PCR product with only the GhUBC1 primer were considered to be transgene-null.

In each BC_1_S_1_ population the numbers of transgenic and null plants indicated the pHellsgate-8::PHYA1 construct segregated as a single locus (i.e., a 3:1 ratio of transgenic to null plants) (**Table 2**). In this study, PCR product peak heights and areas were highly correlated for the 35S_S and OCS_S primer pairs, indicating similar efficiencies in target site amplification (**Table 3**). In contrast, NPTII-3 did not amplify consistently across all BC_1_S_1_ populations and its product characteristics were not correlated with those of 35S_S and OCS_S (**Table 3**).

**Table 2:** Segregation of the pHellsgate-8::PHYA1 construct in nine BC_1_S_1_ populations.

BC_1_S_1_ population	Transgenic plants^a^	Null plants^b^	Missing data^c^	Total
Coker312	160	68	12	240
DP90	158	66	16	240
SG747	171	48	21	240
PSC355	162	56	22	240
FM966	174	60	6	240
UA222	170	66	4	240
TAM B182-33	179	56	5	240
MD51ne	181	49	10	240
MD90ne	159	58	23	240
**Total**	**1514**	**527**	**119**	**2160**


*^a^Successful amplification of the 35S_S, OCS_S, and NPTII-3 target sequences.*

*^b^Successful amplification of only the GhUBC1 target sequence. ^c^Missing due to non-amplification or aberrant amplification of target sequences.*

**Table 3:** Correlations between 35S, OCS, and NPTII PCR product peak heights and peak areas.

	OCS height	NPTII height	35S area	OCS area	NPTII area
35S height	0.849^∗^	0.492	0.991^∗^	0.835^∗^	0.497
OCS height		0.426	0.821^∗^	0.933^∗^	0.423
NPTII height			0.480	0.392	0.997^∗^
35S area				0.825^∗^	0.492
OCS area					0.395


*^∗^Statistically significant at *P* ≤ 0.05.*

**FIGURE 3:**
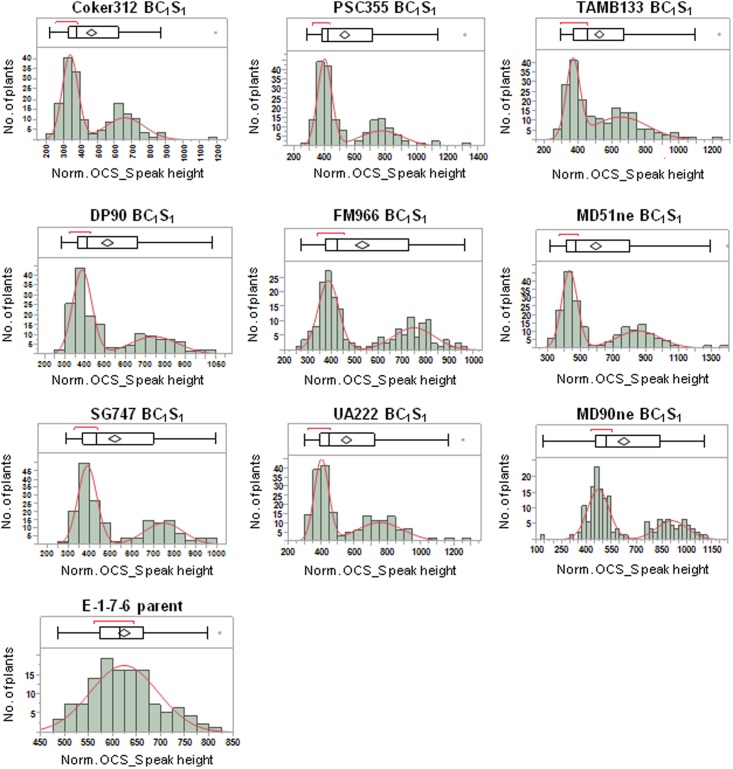
Distributions of normalized OCS_S PCR product peak height values for nine BC_1_S_1_ populations derived from crosses between non-transgenic cultivars and the E-1-7-6 line homozygous for the pHellsgate-8::PHYA1 RNAi construct. The boxplot above each histogram shows selected quantiles of continuous distributions and outliers.

The successful discrimination between transgene homozygous and hemizygous plants would manifest as a bimodal distribution of normalized PCR product values in a BC_1_S_1_ population where each peak in the distribution would represent a distinct genotype. A bimodal distribution is a mixture of two normal distributions with means (μ1 and μ2) and standard deviations (σ1 and σ2); therefore, the distributions of normalized PCR product peak height and area values for 35S_S, OCS_S, and NPTII-3 were analyzed for each of the nine BC_1_S_1_ populations. A clear bimodal distribution of normalized peak height values was readily apparent for the OCS_S primer in each BC_1_S_1_ population (**Figure 3**). In each population a clear demarcation between the mode peaks is observed. This demarcation is observed as a region of low observation frequency and is called the ‘pit.’ The pit provides a natural split point to cluster the homozygous and hemizygous genotypic classes. In contrast to the BC_1_S_1_ populations, a survey of 125 E-1-7-6 plants showed a normal distribution of normalized OCS_S peak heights (**Figure 3**). The 35S_S and NPTII-3 primers did not produce clear bimodal distributions for each BC_1_S_1_ population (data not shown). This was most likely due to decreased robustness in PCR efficiency of these primers from sample to sample.

**FIGURE 4:**
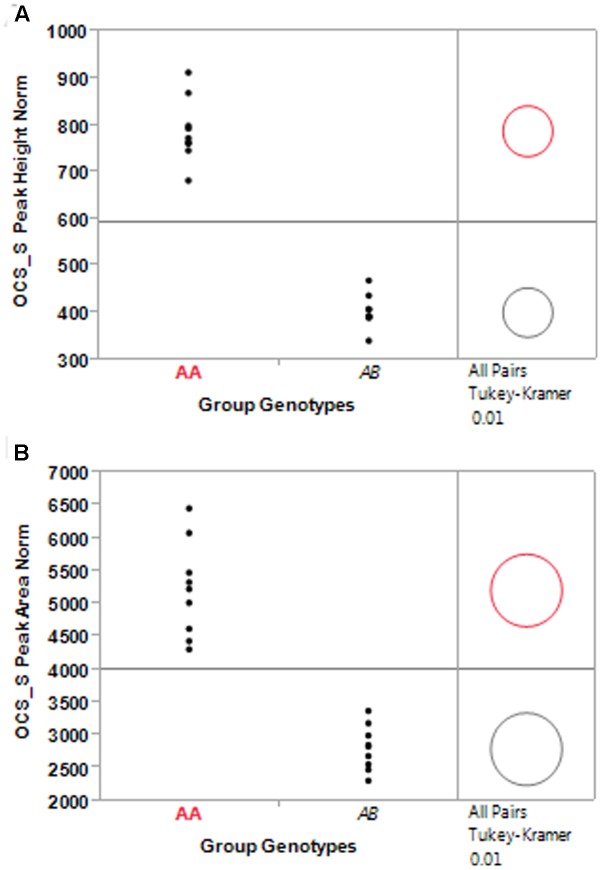
Significance test of sub-population means between homozygous (Group Genotype AA) and hemizygous (Group Genotype AB) entries identified by K-means clustering of normalized OCS_S PCR product peak height **(A)** and peak area **(B)** for nine BC_1_S_1_ populations.

In addition to using the bimodal natural split to cluster entries in each population, a K-Means clustering analysis with *k* = 2 was conducted as well (**Table 4**). The criterion function of the K-means algorithm is the squared distance of the data items from their nearest cluster centroids. The mean normalized OCS_S PCR product peak height, across all nine BC_1_S_1_ populations, was 784.6 for the homozygous group and 406.1 for the hemizygous group. A similar relationship was observed for the mean normalized peak area (homozygous = 5182.4; hemizygous = 2822.1) (**Table 4**). When analyzed individually, the grouping of the population peak height and area means according to homozygous and hemizygous status was statistically significant (**Figure 4**). Out of 1514 plants classified as transgenic, we found only 45 instances where the bimodal natural split and K-Means clustering methods disagreed in identifying a particular sample as homozygous or hemizygous. Using the K-Means clustering analysis to identify transgene homozygous and hemizygous plant allowed us to determine that the pHellsgate-8::PHYA1 construct segregated as a single locus in each BC_1_S_1_ population. In each population the numbers of homozygous, hemizygous, and transgene-null plants fit a 1:2:1 ratio that was significant according to Chi-square analysis (data not shown).

**Table 4:** Transgene zygosity in BC_1_S_1_ populations determined by K-means clustering of normalized OCS_S PCR product peak heights and areas.

	Homozygous			Hemizygous			
			
Population	No. plants	Peak height	Peak area	No. plants	Peak height	Peak area	^a^CCC
Coker312	57	678.2	4426.7	103	344.4	2315.4	3.57
DP90	50	766.6	4587.8	108	389.7	2556.1	7.32
SG747	58	757.7	4271.2	113	394.0	2471.4	8.31
PSC355	52	796.7	5177.6	110	405.6	2676.8	4.22
FM966	64	760.0	5296.2	110	395.4	2868.4	7.76
UA222	62	788.6	5461.5	108	414.2	3044.1	4.63
TAM B182-33	63	740.1	4961.6	116	405.4	2885.3	2.02
MD51ne	66	862.3	3018.0	115	436.7	3190.3	6.93
MD90ne	55	910.9	6440.9	104	469.2	3390.7	8.26
**Average**	**59**	**784.6**	**5182.4**	**110**	**406.1**	**2822.1**	**5.89**


*^a^Cubic Clustering Criterion was used to evaluate model (CCC ≥ 2.00 means model is good fit for data).*

### Confirmation of Transgenic BC_1_S_1_ Zygosity in BC_1_S_2_ Progeny

**FIGURE 5:**
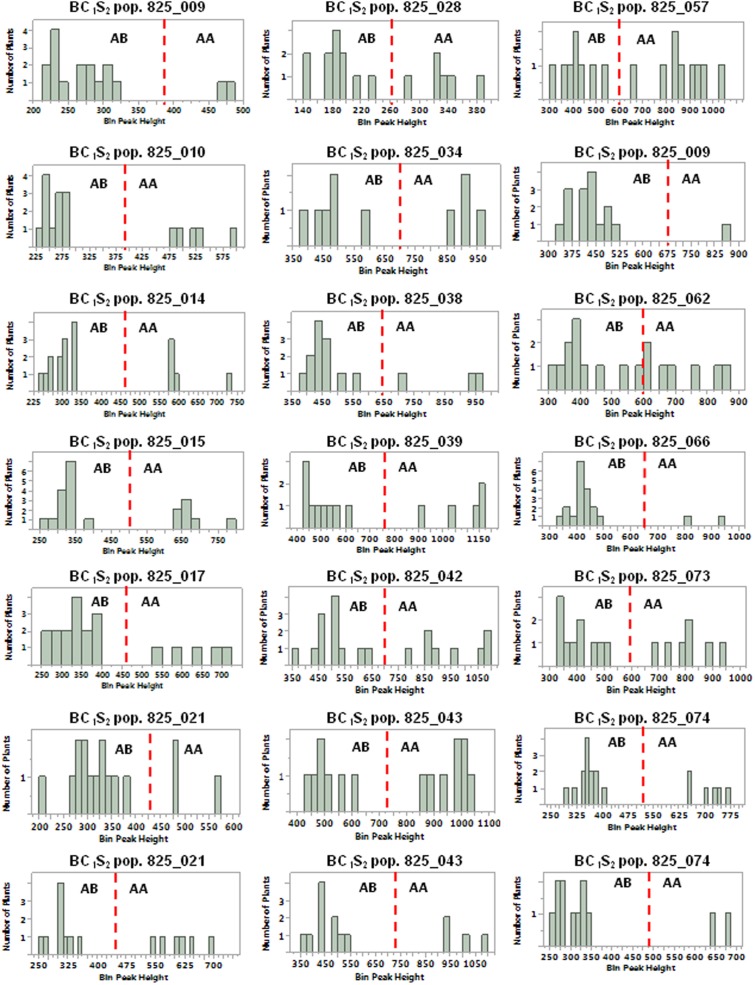
Distribution of normalized OCS_S PCR product height values for BC_1_S_2_ populations derived from BC_1_S_1_ plants predicted to be hemizygous for the pHellsgate-8::PHYA1 RNAi construct. The red-dotted line in each histogram demarcates between hemizygous (AB) and homozygous (AA) progeny plants in each BC_1_S_2_ population.

BC_1_S_1_ plants identified as transgene homozygous or hemizygous were self-fertilized to produce BC_1_S_2_ populations for progeny testing with the 35S_S, OCS_S, and NPTII-3 primers. Of 20 homozygous BC_1_S_1_ plants selected, only one showed progeny that segregated for the pHellsgate-8::PHYA1 construct with the remaining 19 BC_1_S_2_ populations showing no construct segregation (**Table 5**). Of 21 BC_1_S_1_ plants identified as hemizygous, all 21 BC_1_S_2_ populations showed a 1:2:1 segregation of the pHellsgate-8::PHYA1 construct (**Table 5**). Furthermore, we again observed the roughly double in value of normalized peak height and area values for homozygous plants versus hemizygous plants (Supplementary Table S4). When the normalized OCS_S product peak height values of each individual BC_1_S_2_ population were visualized by histogram, a bimodal distribution pattern was observed indicating the presence of homozygous and hemizygous plants (**Figure 5**). Almost without exception, a clear separation was observed between the transgene homozygous and hemizygous groups (**Figure 5**). These results demonstrate a predictive success of 95% for transgene homozygous BC_1_S_1_ plants and 100% for hemizygous plants.

**Table 5:** Confirmation of BC_1_S_1_ transgene genotype prediction in BC_1_S_2_ progeny families.

Predicted BC_1_S_1_ plant genotype	No. of selfed BC_1_S_1_ plants	No. of correct BC_1_S_1_ predictions	^a^Homozygous BC_1_S_2_ plants/family	^a^Hemizygous BC_1_S_2_ plants/family	^a^Null BC_1_S_2_ plants/family
Homozygous	20	19	22.5	0.0	0.0
Hemizygous	21	21	5.0	11.6	6.3


*^a^Mean number per self-fertilized BC_1_S_1_ plant. Homozygous and hemizygous classifications determined by K-means clustering of normalized OCS_S PCR product peak height values.*

## Conclusion

Biotechnology holds great promise in overcoming many obstacles that face modern agriculture. Observing the behavior of a particular transgene in different genetic backgrounds of the target crop is a major step in trait development. Toward that end, the discrimination between homozygous and hemizygous plants is essential. The method described in this report accomplishes this task by taking advantage of the same principles that underlie quantitative PCR; however, unlike qPCR, our method does not require specialized instrumentation or expensive consumables and can be readily implemented by crop breeding laboratories.

## Author Contributions

JJ, MW, and DD planned the experiment. LG, DD, and JM collected data. LG, DD, and MW interpreted data. LG, DD, and MW wrote the manuscript. IA developed and provided the original E-1-7-6 transgenic line.

## Disclaimer

Mention of trade names or commercial products in this article is solely for the purpose of providing specific information and does notimply recommendation or endorsement by the United States Department of Agriculture.

## Conflict of Interest Statement

The authors declare that the research was conducted in the absence of any commercial or financial relationships that could be construed as a potential conflict of interest.
