# Supplementary material for: A High-Throughput Standard PCR-Based Genotyping Method for Determining Transgene Zygosity in Segregating Plant Populations
Source: Front Plant Sci. 2017 Jul 24;8:1252. doi: 10.3389/fpls.2017.01252 (PMC5522864; doi:10.3389/fpls.2017.01252)
Supplement: Supplementary file 1 [file Table_1.DOCX]

**Table S1.** Homozygous and hemizygous identification efficiency under different genotyping operation parameters. Example: “3.0mM, NO, 10, 05X” = 3.0 mM MgCl_2_, No multiplex PCR of 4 primers, 10 cycles of PCR amplification, 5x dilution of PCR products before capillary electrophoresis.

| **Parameters** | **Summary of Homozygous and Hemizygous Identification Efficiency** | ***OCS_S Peak Height %** | ***35S_S Peak Height %** | ***NPTII-3 Peak Height %** | **Mean** |
| --- | --- | --- | --- | --- | --- |
| 3.0mM, No, 10, 05x | Separation well for Homozygous, Hemizygous, and Null | 65.2% | 40.8% | 51.7% | 52.6% |
| 3.0mM, No, 10, 15x | Separation well for Homozygous, Hemizygous, and Null | 66.2% | 43.1% | 50.4% | 53.3% |
| 3.0mM, No, 20, 05x | Low peak generated from NPTII in the Coker 312 and one Null | 106.0% | 113.0% | 102.1% | 107.0% |
| 3.0mM, No, 20, 15x | Low peak generated from NPTII in the Coker 312 and one Null | 95.5% | 119.0% | 97.7% | 104.0% |
| 3.0mM, No, 30, 05x | Low peak generated from NPTII and OCS in the 2 Nulls | 86.3% | 97.4% | 97.8% | 93.8% |
| 3.0mM, No, 30, 15x | Low peak generated from NPTII and OCS in the 2 Nulls | 98.6% | 94.2% | 99.2% | 97.3% |
| 3.0mM, No, 40, 05x | Low peak generated from NPTII in the 2 Nulls and Coker 312 | 89.7% | 99.7% | 100.9% | 96.8% |
| 3.0mM, No, 40, 15x | Low peak generated from NPTII in the 2 Nulls and Coker 312 | 151.1% | 115.6% | 107.4% | 124.7% |
| 3.0mM, Yes, 10, 05x | Separation well for Homozygous, Hemizygous, and Null | 47.8% | 49.9% | 47.3% | 48.3% |
| 3.0mM, Yes, 10, 15x | Separation well for Homozygous, Hemizygous, and Null | 41.9% | 47.2% | 42.1% | 43.8% |
| 3.0mM, Yes, 20, 05x | Low peak generated from NPTII in one Null | 71.0% | 84.5% | 83.0% | 79.5% |
| 3.0mM, Yes, 20, 15x | Low peak generated from NPTII in one Null | 64.6% | 74.6% | 66.6% | 68.6% |
| 3.0mM, Yes, 30, 05x | Low peak generated from NPTII in 2 Nulls and Coker 312 | 106.2% | 87.3% | 94.7% | 96.1% |
| 3.0mM, Yes, 30, 15x | Low peak generated from NPTII in 2 Nulls | 80.5% | 84.5% | 81.5% | 82.2% |
| 3.0mM, Yes, 40, 05x | Low peak generated from NPTII and OCS in one Null and Coker 312 | 103.9% | 87.4% | 99.6% | 97.0% |
| 3.0mM, Yes, 40, 15x | Low peak generated from NPTII and OCS in one Null and Coker 312 | 97.7% | 80.8% | 84.7% | 87.7% |
| 3.5mM, Yes, 10, 05x | Separation well for Homozygous, Hemizygous, and Null | 53.0% | 50.6% | 52.6% | 52.1% |
| 3.5mM, Yes, 10, 15x | Separation well for Homozygous, Hemizygous, and Null | 53.4% | 52.2% | 51.8% | 52.5% |
| 3.5mM, Yes, 20, 05x | Low peak generated from NPTII in one Null | 101.2% | 76.1% | 97.2% | 91.5% |
| 3.5mM, Yes, 20, 15x | Low peak generated from NPTII in one Null | 121.0% | 111.2% | 121.4% | 117.9% |
| 3.5mM, Yes, 30, 05x | Low peak generated from NPTII in one Null and Coker 313 | 96.7% | 84.9% | 99.5% | 93.7% |
| 3.5mM, Yes, 30, 15x | Low peak generated from NPTII in one Null and Coker 312 | 102.4% | 82.9% | 97.0% | 94.1% |
| 3.5mM, Yes, 40, 05x | Low peak generated from NPTII in one Null and 35S in Coker 312 | 96.7% | 108.8% | 98.4% | 101.3% |
| 3.5mM, Yes, 40, 15x | Low peak generated from NPTII in one Null | 81.5% | 76.9% | 77.2% | 78.5% |
| 3.0mM, Yes, 05, 01x | NO Data |  |  |  |  |
| 3.0mM, Yes, 05, 05x | NO Data |  |  |  |  |
| 3.0mM, Yes, 05, 10x | NO Data |  |  |  |  |
| 3.0mM, Yes, 10, 01x | Separation well for Homozygous, Hemizygous, and Null | 40.3% | 40.1% | 41.0% | 40.6% |
| 3.0mM, Yes, 10, 05x | Separation well for Homozygous, Hemizygous, and Null | 48.9% | 47.1% | 52.1% | 49.6% |
| 3.0mM, Yes, 10, 10x | Separation well for Homozygous, Hemizygous, and Null | 50.0% | 50.7% | 50.6% | 50.6% |
| 3.0mM, Yes, 15, 01x | Low peak generated from 3 Primers in the Null and Coker 312 | 61.3% | 63.3% | 69.0% | 66.2% |
| 3.0mM, Yes, 15, 05x | Low peak generated from 3 Primers in the Null and Coker 312 | 81.5% | 74.0% | 68.7% | 71.4% |
| 3.0mM, Yes, 15, 10x | Low peak generated from 3 Primers in the Null and Coker 312 | 49.4% | 49.6% | 39.4% | 44.5% |
| 3.5mM, Yes, 05, 01x | NO Data |  |  |  |  |
| 3.5mM, Yes, 05, 05x | NO Data |  |  |  |  |
| 3.5mM, Yes, 05, 10x | NO Data |  |  |  |  |
| 3.5mM, Yes, 10, 01x | Separation well for Homozygous, Hemizygous, and Null | 53.1% | 47.6% | 50.3% | 48.9% |
| 3.5mM, Yes, 10, 05x | Separation well for Homozygous, Hemizygous, and Null | 49.6% | 50.8% | 50.2% | 50.5% |
| 3.5mM, Yes, 10, 10x | Separation well for Homozygous, Hemizygous, and Null | 56.5% | 54.1% | 57.6% | 55.8% |
| 3.5mM, Yes, 15, 01x | Low peak generated from 3 Primers in the Null and Coker 312 | 64.2% | 70.4% | 84.2% | 77.3% |
| 3.5mM, Yes, 15, 05x | Low peak generated from 3 Primers in the Null and Coker 312 | 57.6% | 68.3% | 60.3% | 64.3% |
| 3.5mM, Yes, 15, 10x | Low peak generated from 3 Primers in the Null and Coker 312 | 82.2% | 90.4% | 88.7% | 89.6% |
| 4.0mM, Yes, 05, 01x | NO Data |  |  |  |  |
| 4.0mM, Yes, 05, 05x | NO Data |  |  |  |  |
| 4.0mM, Yes, 05, 10x | NO Data |  |  |  |  |
| 4.0mM, Yes, 10, 01x | Separation well for Homozygous, Hemizygous, and Null | 38.0% | 39.7% | 37.1% | 38.4% |
| 4.0mM, Yes, 10, 05x | Separation well for Homozygous, Hemizygous, and Null | 47.6% | 43.7% | 48.1% | 45.9% |
| 4.0mM, Yes, 10, 10x | Separation well for Homozygous, Hemizygous, and Null | 69.6% | 69.4% | 67.0% | 68.2% |
| 4.0mM, Yes, 15, 01x | Low peak generated from 3 Primers in the Null and Coker 312 | 60.4% | 78.3% | 61.5% | 69.9% |
| 4.0mM, Yes, 15, 05x | Low peak generated from 3 Primers in the Null and Coker 312 | 70.2% | 82.8% | 73.7% | 78.2% |
| 4.0mM, Yes, 15, 10x | Low peak generated from 3 Primers in the Null and Coker 312 | 63.8% | 85.6% | 66.2% | 75.9% |

*(Hemizygous peak height / Homozygous peak height) × 100
