# Supplementary material for: A High-Throughput Standard PCR-Based Genotyping Method for Determining Transgene Zygosity in Segregating Plant Populations
Source: Front Plant Sci. 2017 Jul 24;8:1252. doi: 10.3389/fpls.2017.01252 (PMC5522864; doi:10.3389/fpls.2017.01252)
Supplement: Supplementary file 2 [file Table_2.DOCX]

| **Table S2.** PCR product peak height and peak area differentiation between transgene homozygous and hemizygous plants using different primers and varied genotyping operation parameters. Example: “3.0mM, NO, 10, 15X” = 3.0 mM MgCl2, no multiplex PCR of 4 primers, 10 cycles of PCR amplification, and 15x dilution of PCR products before capillary electrophoresis. | | | | | | | | |
| --- | --- | --- | --- | --- | --- | --- | --- | --- |
| **Primer** | **PCR Product Measurement** | **Sample Name** | **3.0 mM, NO, 10, 15X** | **3.0 mM, NO, 10, 5X** | **3.0 mM, YES, 10, 15X** | **3.0 mM, YES, 10, 5X** | **3.5 mM, YES, 10, 15X** | **3.5 mM, YES, 10, 5X** |
| 35S_S | Peak Area | Coker 312 x E-1-7-6 | 283 | 505 | 264 | 544 | 484 | 855 |
|  |  | E-1-7-6 | 640 | 1,233 | 564 | 1,081 | 928 | 1,709 |
|  | Peak Height | Coker 312 x E-1-7-6 | 58 | 104 | 55 | 110 | 103 | 178 |
|  |  | E-1-7-6 | 134 | 255 | 117 | 220 | 198 | 352 |
| NPTII-3 | Peak Area | Coker 312 x E-1-7-6 | 948 | 1,862 | 634 | 1,311 | 993 | 1,775 |
|  |  | E-1-7-6 | 1,950 | 3,598 | 1,466 | 2,859 | 1,928 | 3,546 |
|  | Peak Height | Coker 312 x E-1-7-6 | 171 | 332 | 109 | 235 | 177 | 322 |
|  |  | E-1-7-6 | 338 | 641 | 258 | 496 | 341 | 611 |
| OCS_S | Peak Area | Coker 312 x E-1-7-6 | 1,048 | 1,997 | 598 | 1,202 | 971 | 1,789 |
|  |  | E-1-7-6 | 1,604 | 2,991 | 1,330 | 2,643 | 1,912 | 3,421 |
|  | Peak Height | Coker 312 x E-1-7-6 | 206 | 360 | 105 | 221 | 195 | 325 |
|  |  | E-1-7-6 | 311 | 553 | 251 | 462 | 364 | 613 |
| GhUBC1 | Peak Area | Coker 312 x E-1-7-6 | 253 | 481 | 107 | 238 | 277 | 477 |
|  |  | E-1-7-6 | 347 | 580 | 112 | 310 | 241 | 444 |
|  | Peak Height | Coker 312 x E-1-7-6 | 42 | 82 | 24 | 40 | 48 | 93 |
|  |  | E-1-7-6 | 50 | 104 | 22 | 37 | 41 | 75 |
