# Supplementary material for: A High-Throughput Standard PCR-Based Genotyping Method for Determining Transgene Zygosity in Segregating Plant Populations
Source: Front Plant Sci. 2017 Jul 24;8:1252. doi: 10.3389/fpls.2017.01252 (PMC5522864; doi:10.3389/fpls.2017.01252)
Supplement: Supplementary file 3 [file Table_3.DOCX]

| **Table S3.** PCR product peak height and peak area differentiation between transgene homozygous and hemizygous genotypes using different primers and varied genotyping operation parameters within a multiplex PCR reaction containing all four primers. Example: “3.0 mM, 10, 10X” = 3.0 mM MgCl_2_, 10 cycles of PCR amplification, and 10x dilution of PCR products before capillary electrophoresis. | | | | | | | | | | | |
| --- | --- | --- | --- | --- | --- | --- | --- | --- | --- | --- | --- |
| **Primer** | **PCR Product Measurements** | **Sample Name** | **3.0 mM, 10, 10X** | **3.0 mM, 10, 01X** | **3.0 mM, 10, 05X** | **3.5 mM, 10, 10X** | **3.5 mM, 10, 01X** | **3.5 mM, 10, 05X** | **4.0 mM, 10, 10X** | **4.0 mM, 10, 01X** | **4.0 mM, 10, 05X** |
| 35S_S | Peak Area | Coker312 x E-1-7-6 | 304 | 860 | 572 | 637 | 1606 | 905 | 411 | 1019 | 781 |
|  |  | DP 90 x E-1-7-6 | 266 | 891 | 399 | 452 | 1057 | 750 | 393 | 1121 | 603 |
|  |  | E-1-7-6 | 578 | 2225 | 1041 | 1025 | 2830 | 1584 | 586 | 2796 | 1568 |
|  | Peak Height | Coker312 x E-1-7-6 | 62 | 161 | 111 | 133 | 304 | 173 | 85 | 195 | 154 |
|  |  | DP 90 x E-1-7-6 | 53 | 171 | 81 | 87 | 208 | 147 | 83 | 215 | 122 |
|  |  | E-1-7-6 | 114 | 414 | 204 | 204 | 538 | 315 | 121 | 517 | 316 |
| NPTII-3 | Peak Area | Coker312 x E-1-7-6 | 788 | 2339 | 1574 | 1364 | 3478 | 1855 | 847 | 2059 | 1643 |
|  |  | DP 90 x E-1-7-6 | 734 | 2530 | 1182 | 1107 | 2767 | 1833 | 859 | 2320 | 1392 |
|  |  | E-1-7-6 | 1554 | 6008 | 2604 | 2212 | 6278 | 3784 | 1293 | 5948 | 3132 |
|  | Peak Height | Coker312 x E-1-7-6 | 138 | 387 | 271 | 237 | 614 | 323 | 150 | 358 | 283 |
|  |  | DP 90 x E-1-7-6 | 132 | 440 | 200 | 193 | 471 | 310 | 143 | 411 | 235 |
|  |  | E-1-7-6 | 267 | 1008 | 452 | 374 | 1080 | 631 | 219 | 1038 | 539 |
| OCS_S | Peak Area | Coker312 x E-1-7-6 | 742 | 2204 | 1579 | 1409 | 3879 | 2007 | 830 | 2202 | 1733 |
|  |  | DP 90 x E-1-7-6 | 744 | 2657 | 1146 | 1071 | 2925 | 1943 | 858 | 2575 | 1371 |
|  |  | E-1-7-6 | 1539 | 6192 | 2601 | 2215 | 6622 | 3896 | 1195 | 6481 | 3014 |
|  | Peak Height | Coker312 x E-1-7-6 | 135 | 341 | 248 | 261 | 598 | 323 | 153 | 307 | 268 |
|  |  | DP 90 x E-1-7-6 | 139 | 420 | 208 | 201 | 467 | 318 | 152 | 389 | 236 |
|  |  | E-1-7-6 | 274 | 944 | 466 | 409 | 1002 | 646 | 219 | 916 | 529 |
| GhUBC1 | Peak Area | Coker312 x E-1-7-6 | 227 | 687 | 414 | 520 | 1145 | 598 | 198 | 499 | 356 |
|  |  | DP 90 x E-1-7-6 | 216 | 814 | 339 | 344 | 867 | 569 | 203 | 517 | 305 |
|  |  | E-1-7-6 | 297 | 952 | 417 | 403 | 1039 | 639 | 250 | 664 | 348 |
|  | Peak Height | Coker312 x E-1-7-6 | 36 | 111 | 70 | 79 | 194 | 105 | 33 | 82 | 65 |
|  |  | DP 90 x E-1-7-6 | 37 | 122 | 54 | 53 | 146 | 91 | 30 | 78 | 48 |
|  |  | E-1-7-6 | 46 | 153 | 74 | 67 | 177 | 107 | 35 | 108 | 59 |
