# Supplementary material for: A High-Throughput Standard PCR-Based Genotyping Method for Determining Transgene Zygosity in Segregating Plant Populations
Source: Front Plant Sci. 2017 Jul 24;8:1252. doi: 10.3389/fpls.2017.01252 (PMC5522864; doi:10.3389/fpls.2017.01252)
Supplement: Supplementary file 4 [file Table_4.DOCX]

| **Table S4.** Transgene segregation in BC_1_S_2_ families derived from hemizygous BC_1_S_1_ plants using normalized OCS_S product values. | | | | | | | | | | |
| --- | --- | --- | --- | --- | --- | --- | --- | --- | --- | --- |
| **BC_1_S_1_ Plants** | | | ***Homozygous BC_1_S_2_ Progeny** | | | ***Hemizygous BC_1_S_2_ Progeny** | | | **Number of transgene-null plants** | **Number of failed PCR reactions** |
| Plant Number | Norm. Peak Height | Norm. Peak Area | Number of Plants | Norm. Peak Height | Norm. Peak Area | Number of Plants | Norm. Peak Height | Norm. Peak Area |  |  |
| 825_017 | 356 | 2,712 | 5 | 631 | 4208 | 15 | 327 | 2144 | 4 |  |
| 825_042 | 97 | 633 | 8 | 951 | 6426 | 12 | 504 | 3497 | 4 |  |
| 825_045 | 380 | 2,353 | 4 | 984 | 7609 | 10 | 456 | 3458 | 10 |  |
| 825_009 | 363 | 2,407 | 2 | 480 | 2929 | 15 | 262 | 1679 | 7 |  |
| 825_010 | 403 | 2,732 | 5 | 525 | 3096 | 12 | 259 | 1694 | 7 |  |
| 825_014 | 430 | 2,531 | 5 | 614 | 4308 | 13 | 297 | 2208 | 5 | 1 |
| 825_015 | 453 | 3,093 | 7 | 676 | 4207 | 14 | 325 | 2155 | 3 |  |
| 825_021 | 457 | 2,520 | 3 | 512 | 3601 | 13 | 308 | 2133 | 8 |  |
| 825_022 | 460 | 2,786 | 6 | 613 | 4033 | 9 | 307 | 1987 | 9 |  |
| 825_028 | 545 | 3,127 | 6 | 333 | 2254 | 11 | 185 | 1176 | 5 | 2 |
| 825_034 | 380 | 2,685 | 4 | 909 | 6514 | 6 | 475 | 3027 | 6 |  |
| 825_038 | 545 | 3,359 | 3 | 875 | 5957 | 12 | 455 | 2999 | 1 |  |
| 825_039 | 513 | 2,881 | 5 | 1089 | 6761 | 9 | 498 | 3364 | 10 |  |
| 825_043 | 361 | 2,560 | 8 | 968 | 6287 | 7 | 503 | 3688 | 9 |  |
| 825_057 | 329 | 2,378 | 9 | 871 | 5181 | 8 | 424 | 2900 | 7 |  |
| 825_059 | 420 | 2,683 | 1 | 862 | 6448 | 15 | 423 | 3094 | 7 | 1 |
| 825_062 | 456 | 3,043 | 7 | 720 | 4726 | 11 | 412 | 2690 | 4 | 2 |
| 825_066 | 361 | 2,129 | 2 | 875 | 5805 | 18 | 420 | 2884 | 4 |  |
| 825_073 | 334 | 2,360 | 7 | 811 | 5980 | 10 | 403 | 3003 | 7 |  |
| 825_074 | 305 | 2,255 | 6 | 711 | 5547 | 13 | 356 | 2850 | 5 |  |
| 825_076 | 384 | 2,300 | 2 | 662 | 4395 | 10 | 298 | 2048 | 11 | 1 |
| Total |  |  | 105 |  |  | 243 |  |  | 133 |  |
| Mean | 397 | 2,549 | 5.0 | 746 | 5,061 | 11.6 | 376 | 2,604 | 6.3 | 0.3 |
| *Determined by K-means clustering. | | | | | | | | | | |
